# Preciseness of artificial intelligence for lateral cephalometric measurements

**DOI:** 10.1007/s00056-023-00459-1

**Published:** 2023-03-09

**Authors:** Mostafa El-Dawlatly, Khaled Hazem Attia, Ahmed Yehia Abdelghaffar, Yehya Ahmed Mostafa, Mohamed Abd El-Ghafour

**Affiliations:** 1 Department of Orthodontics, Faculty of Dentistry, Cairo University, 11 El-Saraya Street, Manial, Cairo, Egypt; 2 Department of Orthodontics, Faculty of Dentistry, Future University, New Cairo, Egypt

**Keywords:** Orthodontics, Artificial intelligence, Lateral cephalometry, Diagnosis, Accuracy, Kieferorthopädie, Künstliche Intelligenz, Fernröntgenseitaufnahme, Diagnose, Genauigkeit

## Abstract

**Background:**

The aim of the study was to assess the accuracy and efficiency of a new artificial intelligence (AI) method in performing lateral cephalometric radiographic measurements.

**Materials and methods:**

A total of 200 lateral cephalometric radiographs were assessed for quality and included. Three methods were used to perform the cephalometric measurements: (1) the AI method using WebCeph software (AssembleCircle Corp., Gyeonggi-do, Republic of Korea), (2) the modified AI method using WebCeph software after manual modification of the landmarks’ position, and (3) using OnyxCeph software (Image Instruments GmbH, Chemnitz, Germany) by manual landmark identification and digital measurements generation. The results of the measurements produced by the three methods were compared, in addition to comparing the time required for the measurements’ generation required for each method.

**Results:**

Statistically significant differences were detected between the measurements resulting from the three used methods. Fewer differences were detected between the modified AI method and the OnyxCeph method. The AI method produced the measurements the fastest followed by the modified AI method and then the OnyxCeph method.

**Conclusions:**

Considering the used AI software, AI followed by manual tuning of the landmarks’ position might be an accurate method in lateral cephalometric analysis. AI alone is still not fully reliable at locating the different landmarks on the lateral cephalometric radiographs.

## Abstract (de)

**Hintergrund:**

Ziel der Studie war es, die Genauigkeit und die Effizienz einer neuen Methode der künstlichen Intelligenz (KI) bei der Durchführung lateraler kephalometrischer radiographischer Messungen zu bewerten.

**Materialien und Methoden:**

Insgesamt wurden 200 seitliche kephalometrische Röntgenaufnahmen auf ihre Qualität hin untersucht und in die Studie aufgenommen. Zur Durchführung der kephalometrischen Messungen wurden 3 Methoden verwendet: (1) die AI-Methode unter Verwendung der WebCeph-Software (AssembleCircle Corp., Gyeonggi-do, Republik Korea), (2) die modifizierte AI-Methode unter Verwendung der WebCeph-Software nach manueller Änderung der Position der Orientierungspunkte und (3) die Verwendung der OnyxCeph-Software (Image Instruments GmbH, Chemnitz, Deutschland) durch manuelle Identifizierung der Orientierungspunkte und Erzeugung digitaler Messungen. Die Ergebnisse der mit den 3 Methoden erstellten Messungen wurden verglichen, ebenso wie die für jede Methode benötigte Zeit zur Erstellung der Messungen.

**Ergebnisse:**

Statistisch signifikante Unterschiede wurden zwischen den mit den 3 verwendeten Methoden erstellten Messungen festgestellt. Zwischen der modifizierten AI-Methode und der OnyxCeph-Methode wurden geringere Unterschiede festgestellt. Bei der AI-Methode wurden die Messungen am schnellsten durchgeführt, gefolgt von der modifizierten AI-Methode und der OnyxCeph-Methode.

**Schlussfolgerungen:**

In Anbetracht der verwendeten KI-Software könnte die KI, gefolgt von einer manuellen Abstimmung der Position der Landmarken, eine genaue Methode für die laterale kephalometrische Analyse sein. KI allein ist immer noch nicht völlig zuverlässig bei der Lokalisierung der verschiedenen Landmarken auf den seitlichen kephalometrischen Röntgenbildern.

## Introduction

Orthodontic data collection and diagnosis is a process that consumes time and effort [1]. This process usually encompasses meticulous analysis of different diagnostic records including photographs, study models, and radiographs [2, 3]. Among these, lateral cephalometrics is one of the most important analyses to be performed before and after treatment [4]. Lateral cephalometric analysis is essential in the process of diagnosis and treatment planning of orthodontic and orthognathic cases to clarify the skeletal and dental relationships, and to analyze growth- and treatment-related changes [2].

Nowadays, the concept of “digital orthodontics” is being increasingly implemented [5]. Recently, the idea of using “artificial intelligence” (AI) in orthodontics has been introduced [6]. Multiple advances utilizing the AI technology have been rapidly implemented in diagnosis and treatment, aiming to fine tune the orthodontic professionality [6]. One of the promising applications of AI in orthodontics is in the field of digital cephalometry [7]. AI was initially incorporated in tracing software as an adjunct that connects the traced points, previously marked by the operator. Then, calculations of angles and distances according to a programmed algorithm would be performed. An innovation was lately released comprising complete automatic detection of different cephalometric landmarks which is nonoperator dependent. Accordingly, identification of all landmarks and the resulting generation of measurements would be completed in seconds [8]. If accurate, this fast production of cephalometric analysis would decrease the time consumed in landmark detection to produce the needed measurements and could reduce the orthodontists’ treatment planning time.

Two previous studies [9, 10] investigated the usage of AI in orthodontics. None of those studies discussed the accuracy of AI in generating the basic cephalometric measurements.

The aim of the current study was to investigate the accuracy and efficiency of AI applying a newly proposed, fully automated tracing software WEB CEPH (AssembleCircle Corp., Gyeonggi-do, Republic of Korea) with and without further manual support in cephalometric measurements production.

## Materials and methods

### Selection of the lateral cephalometric radiographs

The sample comprised pretreatment lateral cephalograms of 200 growing and adolescent female patients that were filtered from 1879 radiographs from the pretreatment records saved at the outpatient clinic computer of the department of orthodontics of two different universities. It was ensured that both universities use the same cephalometric radiograph equipment to avoid different magnification. All radiographs used in the current study were meticulously selected to be free from any artifacts. Clear anatomical landmarks, presence of a ruler for calibration, patient in natural head position, and shadows of the cephalostat and mandible in centric relation were the criteria upon which the radiographs were checked for eligibility to be included in the current study.

### Measurements’ production

**Fig. 1:**
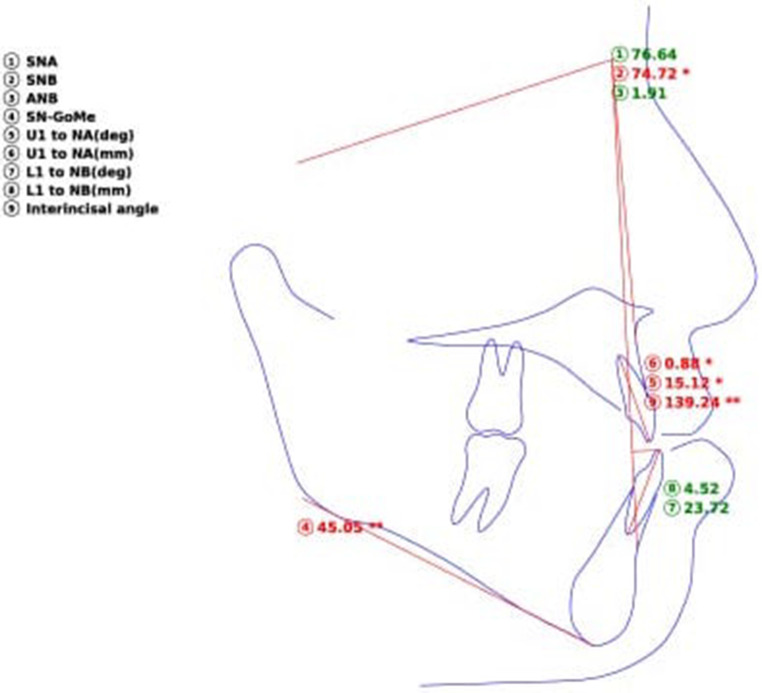
Lateral cephalometric measurements Laterale kephalometrische Messungen

**Fig. 2:**
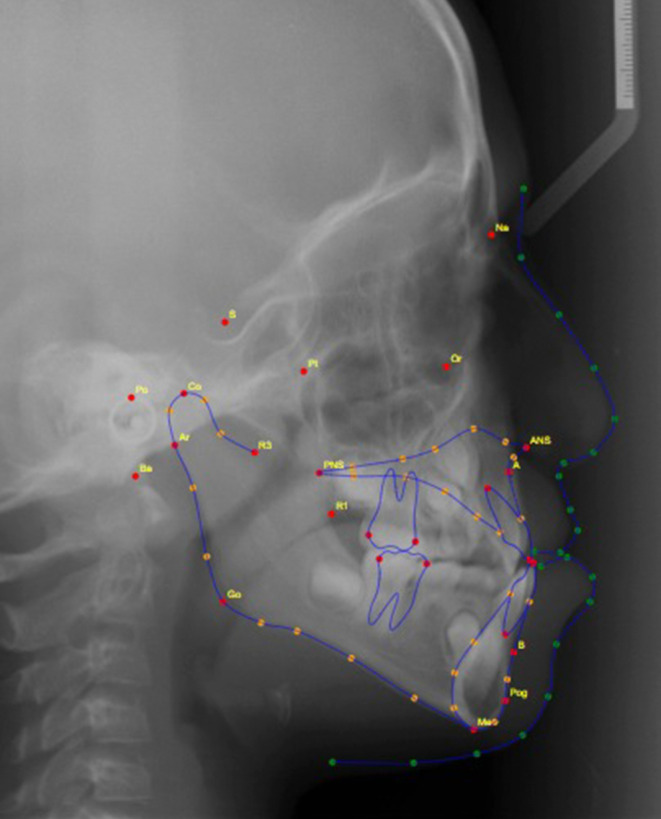
Lateral cephalometric radiograph with the fully automated landmark identification and tracing Laterale kephalometrische Aufnahme mit vollautomatischer Identifizierung und Durchzeichnung von Referenzpunkten

**Fig. 3:**
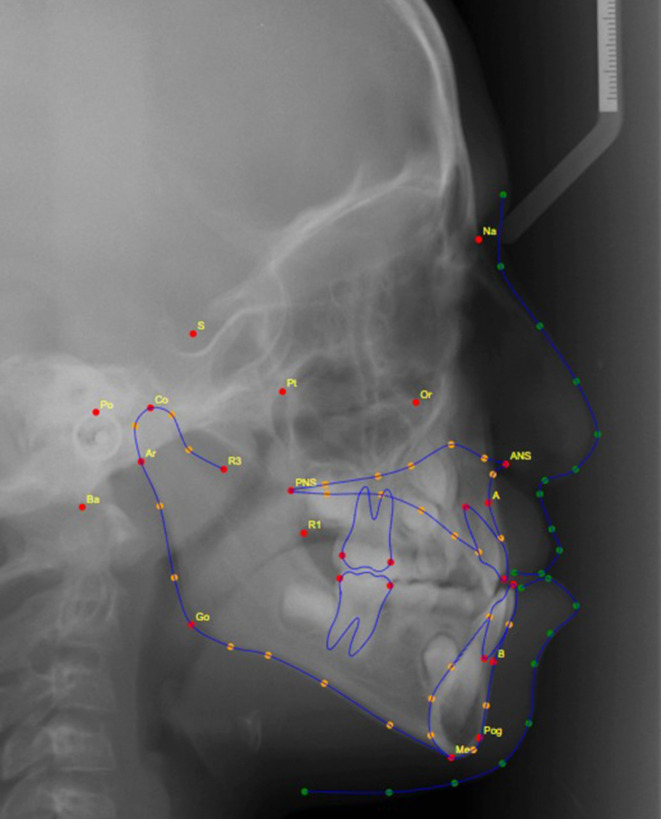
Lateral cephalometric radiograph with the fully automated landmarks identification and tracing followed by manual tuning of the landmark positions Laterale kephalometrische Aufnahme mit vollautomatischer Identifizierung und Durchzeichnung der Referenzpunkte und anschließender Einstellung der Position der Referenzpunkte

After quality checking, all selected radiographs were used to generate the cephalometric measurements mentioned in Table 1 and Fig. 1. The measurements were attained by three methods: (1) using OnyxCeph software (Image Instruments GmbH, Chemnitz, Germany), where manual landmark detection was completed followed by digital calculation of the measurements using the software’s algorithms; (2) using WebCeph [8] website (www.webceph.com) for automatic landmark detection (using AI) and measurements calculation (Fig. 2); and (3) using AI provided by the WebCeph [8] website followed by manual tuning and modifying the automatically located landmarks (Fig. 3).

**Table 1:** Cephalometric measurements and definitions [12] Kephalometrische Messungen und Definitionen [12]

Measurement	Definition
SNA (°)	Angle formed by the SN line and the NA line 1
SNB (°)	Angle formed by the SN line and the NB line 1
ANB (°)	Angle formed by the NA line and the NB line 1
U1 to NA, mm	Distance from incisal edge of upper 1 to N‑B line
U1 to NA (°)	Angle from incisal edge of upper 1 to N‑A line
Interincisal angle (°)	The angle between the long axes of the maxillary and mandibular incisors 1
L1 to NB, mm	Distance from incisal edge of lower 1 to N‑B line
L1 to NB (°)	Angle from incisal edge of lower 1 to N‑B line
Sn to GoMe (°)	Angle formed by the SN line and the Go Me line

The primary outcome was to compare the accuracy of the measurements produced by the fully automated software WEB CEPH (with and without manual modifications) with the measurements derived from the regular tracing software. In addition, counting and comparing the complete analysis time required using the three methods was the secondary outcome.

### Sample size calculation

Sample size calculation was performed using data from a previous study measuring the SNA angle with the help of the OnyxCeph software [11]. The acceptable difference between the SNA angle calculated with OnyxCeph and that produced by the AI was set at 1°. Using the standard deviation of 4.6 from that paper, a power of 80% and a type I error of 0.5 calculation was performed with the PS calculator (version 3.1.2, Creative Commons Attribution-NonCommercial-NoDerivs 3.0, USA). The calculation indicated a need for a minimum of 168 cephalometric radiographs; thus, 200 radiographs were included in the current study.

### Statistics

The significance level was set at *P* ≤ 0.05. Statistical analysis was performed with SPSS® Statistics (version 20, IBM, Armonk, NY, USA). Handling of data was done using Excel software (Microsoft, Redmond, WA, USA).

Data were explored for normality using Kolmogorov–Smirnov and Shapiro–Wilk tests. According to the behavior of the data (either parametric or nonparametric), the suitable statistical test was selected.

The means, standard deviations (SD), and confidence intervals (CI) were calculated for each group in each test. For normally distributed data, one-way analysis of variance (ANOVA) was used to compare the results of the three methods, followed by the Bonferroni test to make comparisons between each of the two methods in each measurement. Due to the general normal distribution of data, nonparametric tests were not used in the current study.

Interclass correlation coefficients (ICC) were calculated to detect the intra- and interobserver reliability of the manual identification of the landmarks in the study.

## Results

All the included 200 radiographs were measured using the three methods. Acceptable intraobserver reliability and agreement between all the readings were found (ICC values ranged from 0.81 to 0.91). For the interobserver reliability, acceptable reliability was also observed for the carried-out measurements (ICC values ranged from 0.79 to 0.92).

**Fig. 4:**
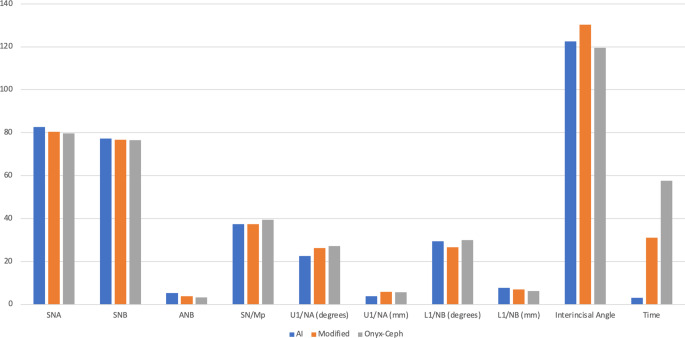
The differences between the methods: *AI* artificial intelligence method, *Modified* the modified artificial intelligence method, *Onyx-Ceph* measurements produced using Onyx-Ceph software (Image Instruments GmbH, Chemnitz, Germany). Abbreviations are defined in Table 1 Die Unterschiede zwischen den Methoden: *AI* KI(Künstliche Intelligenz)-Methode, *Modified* Modifizierte KI-Methode, *Onyx-Ceph* Mit der Software Onyx-Ceph (Image Instruments GmbH, Chemnitz, Deutschland) erstellte Messungen. Die Definitionen der Abkürzungen finden sich in Tab. 1

For the overall comparison between the three methods (Table 2 and Fig. 4), statistically significant differences were found in all measurements and the time required for the measuring process.

**Table 2:** Mean and standard deviation of the different cephalometric measurements by the three methods compared using one-way analysis of variance (ANOVA) Mittelwert und Standardabweichung der verschiedenen kephalomterischen Messungen mit den drei Methoden im Vergleich mittels einseitiger Varianzanalyse (ANOVA)

Measurements	AI		Modified		OnyxCeph		*P*-Value
	Mean	SD	Mean	SD	Mean	SD	
SNA (°)	82.62^a^	3.12	80.39^b^	3.46	79.62^c^	3.85	0.001*
SNB (°)	77.31^a^	3.50	76.64^b^	3.86	76.44^b^	4.10	0.001*
ANB (°)	5.30^a^	3.22	3.74^b^	3.30	3.18^c^	3.45	0.001*
SN/MP (°)	37.37^a^	6.75	37.37^a^	6.27	39.37^b^	6.53	0.001*
U1/NA (°)	22.54^a^	5.37	26.19^ab^	22.75	27.26^b^	8.24	0.001*
U1/NA (mm)	3.84^a^	1.99	5.84^b^	3.30	5.59^b^	3.13	0.001*
L1/NB (°)	29.43^a^	5.74	26.70^b^	10.52	29.97^a^	7.49	0.001*
L1/NB (mm)	7.71^a^	3.40	6.95^b^	2.95	6.17^c^	2.74	0.001*
Interincisal angle (°)	122.57^a^	9.04	130.25^ab^	71.40	119.61^b^	12.17	0.001*
Time (sec)	3.00^a^	0.00	31.07^b^	12.02	57.65^c^	14.55	0.001*

*Significant (*p* < 0.05), *ns* nonsignificant (*p* > 0.05), values sharing the same letter are not statistically significant, values sharing different letters are statistically significant, *SD* standard deviation, *AI* fully automated landmarks identification and tracing using artificial intelligence (AI), *modified* fully automated landmarks identification and tracing followed by tuning of the landmark positions, *OnyxCeph* manual landmark detection completed followed by digital calculation of the measurements using software algorithms

For the pairwise comparisons (Table 3), statistically significant differences were found when comparing the AI vs the modified AI methods for all measurements except for the SN/MP, U1/NA angles, and the interincisal angle which did not differ significantly. When comparing the AI method vs the OnyxCeph analysis, statistically significant differences were detected for all measurements except for the L1/NB angle. For the modified AI method versus the OnyxCeph analysis, statistically significant differences were detected for the SNA, ANB, SN/MP, L1/NB angles, and the L1/NB linear measurement. The other measurements did not differ significantly.

**Table 3:** Mean differences and 95% confidence interval of the different cephalometric measurements between two methods, the Bonferroni test was used to compare two methods in each measurement Mittlere Unterschiede und das 95 %-Konfidenzintervall der verschiedenen kephalometrischen Messungen zwischen 2 Methoden. Zum Vergleich zweier Methoden bei jeder Messung wurde der Bonferroni-Test verwendet

Measurements^a^	AI/Modified			Modified/OnyxCeph			AI/OnyxCeph		
	Mean Diff	95% CI	*P*-value	Mean Diff	95% CI	*P*-value	Mean Diff	95% CI	*P*-value
SNA (°)	2.22	1.82 to 2.61	0.001*	0.77	0.34 to 1.19	0.001*	2.992	2.50 to 3.48	0.001*
SNB (°)	0.66	0.37 to 0.94	0.001*	0.20	−0.09 to 0.51	0.307 ns	0.86	0.50 to 1.23	0.001*
ANB (°)	1.56	1.36 to 1.76	0.001*	0.56	0.27 to 0.85	0.001*	2.12	1.85 to 2.40	0.001*
SN/MP (°)	0.001	−0.51 to 0.51	1 ns	−2	−2.67 to −1.32	0.001*	−1.999	−2.81 to −1.18	0.001*
U1/NA (°)	3.65	−0.06 to 7.36	0.056 ns	1.07	−2.68 to 4.83	1 ns	4.72	3.60 to 5.84	0.001*
U1/NA (mm)	1.99	1.60 to 2.38	0.001*	0.24	−0.22 to 0.70	0.617 ns	1.75	1.32 to 2.17	0.001*
L1/NB (°)	2.723	1.19 to 4.25	0.001*	3.27	1.71 to 4.82	0.001*	0.54	−0.20 to 1.30	0.244 ns
L1/NB (mm)	0.75	0.40 to 1.11	0.001*	0.78	0.58 to 0.98	0.001*	1.54	1.13 to 1.94	0.001*
Interincisal angle (°)	7.68	−4.45 to 19.81	0.384 ns	10.63	−1.42 to 22.69	0.103 ns	2.95	1.60 to 4.30	0.001*
Time (sec)	28.078	26.02 to 30.13	0.001*	26.57	23.93 to 29.21	0.001*	54.65	52.16 to 57.13	0.001*

* significant (*p* < 0.05), *ns* not significant (*p* > 0.05), *CI* confidence interval

^a^Abbreviations defined in Table 1

For the time elapsed in the measuring process (Table 2 and Fig. 4), statistically significant differences were detected between the three methods, denoting that the AI method was the fastest followed by the modified AI method then the measurement using OnyxCeph.

## Discussion

Lateral cephalometric analysis is an integral step in orthodontic diagnosis and treatment planning [4]. The process of point identification and generation of the measurements is very tedious and time consuming. Thus, any accurate attempts to accelerate the process would be of great benefit. Recent technologies and AI provided a new approach for cephalometric landmarks identification and generation of measurements. Testing the accuracy of these technologies is crucial to confirm the reliability of its usage and to provide clinicians with a simpler approach for cephalometric analysis.

The results of the current study are interesting. Although the differences between application of the AI alone and the conventional digital tracing were significant, the modified AI method resulted in readings closer to the conventional method. Based on this, fine tuning by the orthodontic clinician for the automatically located landmarks would be mandatory to achieve accurate final readings. This also makes unnecessary the need for further machine learning of the algorithm to be more precise in locating some cephalometric landmarks.

The current study also helped in detecting the measurements that were most affected by inaccurate localization of the points by the program. This could act as a guideline for the software provider in the process of enhancing the point detection efficiency throughout the learning of the algorithm. Comparing the results of the current study to the results of other studies that tested the efficiency of other fully automated tracing software programs [9, 10], some differences were detected. Both studies [9, 10] found that the AI alone is an accurate tool for cephalometric landmark identification, this was not the case in the current study.

Assessing the total tracing time required by the three different methods was also crucial in the current study. As the main purpose of inventing the fully automated tracing software was to reduce the time required by the clinician to locate the landmarks. The current software proved to be efficient in this aspect, even when the modified version was tested. Both the fully automated AI and the AI with operator modifications required significantly less time than the regular digital tracing method.

## Conclusions

Within the limitations of the current study and also considering the use of AI software from only one provider, the following can be concluded:

- The AI method followed by fine tuning of the location of landmarks (the modified AI method) was successful and can be used as an alternative to ordinary digital landmark identification for lateral cephalometric analysis. The modified AI method was the most efficient method.
- Use of AI alone was not accurate enough for landmark identification and accordingly not precise in the generation of lateral cephalometric measurements.
- Measurements were generated fastest using the AI method, followed by the modified AI method, and finally the conventional digital method.
